# Uncertainty-Aware Machine Learning for Compact-Model-Based Design and Identifiability Analysis of WSe_2_ *p*-Channel Transistors

**DOI:** 10.3390/mi17091070

**Published:** 2026-09-09

**Authors:** Zhengran He, Kyeiwaa Asare-Yeboah, Meng Su, Jie Zhao

**Affiliations:** 1 Electrical and Computer Engineering, Pennsylvania State University Behrend, Erie, PA 16563, USA; 2 Computer Science and Software Engineering, Pennsylvania State University Behrend, Erie, PA 16563, USA

**Keywords:** WSe_2_, field-effect transistor, machine learning, Sobol sensitivity, multiobjective optimization

## Abstract

Machine-learning surrogates can accelerate transistor design, but optimized predictions require physical consistency and explicit treatment of surrogate uncertainty. Here, we develop an uncertainty-aware machine-learning framework for compact-model-based design of WSe_2_ *p*-channel field-effect transistors using an experimentally calibrated S2DS model. A 5000-device dataset spanning channel length, equivalent oxide thickness, hole mobility, contact resistance, impurity density, trap density, and gate-voltage offset was used to train a censor-aware neural-network surrogate for full *p*-branch transfer-curve prediction at two drain biases. On an independent 750-device test set, the surrogate achieved a mean pointwise $R^{2\;}$ of 0.9891 and an exact-coordinate RMSE of 0.0799 decade. The framework further combined inverse-identifiability analysis, Sobol sensitivity analysis, and multiobjective optimization of saturation on-state current, saturation-bias maximum transconductance, normalized drain-bias threshold shift, and threshold-voltage placement. Screening of 262,144 candidate designs yielded 48 Pareto-optimal solutions, of which nine satisfied the uncertainty-screening criteria and six retained all four performance claims after direct S2DS reevaluation. These results demonstrate a physically grounded and uncertainty-aware approach for efficient WSe_2_ transistor design within an experimentally calibrated compact-model domain.

## 1. Introduction

Transition-metal dichalcogenides offer atomically thin channels, strong electrostatic gate control, and device architectures that are well suited to continued scaling beyond conventional bulk semiconductors [1,2]. Among these materials, WSe_2_ is particularly attractive for *p*-channel field-effect transistors (FETs) because of its favorable hole transport [3,4,5], compatibility with high-work-function contacts for efficient hole injection [6,7], and potential role in complementary two-dimensional (2D) logic [8,9,10,11]. However, translating promising individual device demonstrations into useful design rules requires understanding how device and channel geometry [12,13], gate electrostatics [14,15,16], charge transport properties [17,18], contact resistance [19,20], charged impurities [21,22], and interface traps [23,24,25] jointly influence measurable transistor performance. Relevant device metrics include on-state current, transconductance, threshold voltage, drain-bias sensitivity of threshold voltage, subthreshold swing (SS), and on/off current ratio. WSe_2_ can exhibit either predominantly unipolar or ambipolar transport depending on contact configuration, electrostatics, and the accessible bias range [26]. The present study intentionally focuses on the *p*-channel branch of WSe_2_ transistor operation. Recent WSe_2_ device concepts, including room-temperature impact-ionization FETs based on stepwise homojunctions, further demonstrate the expanding range of WSe_2_-based transistor architectures [27]. Systematically exploring the coupled dependence of these performance metrics on multiple device parameters experimentally remains costly and time-consuming.

Physics-based compact modeling [28,29] provides an efficient and physically interpretable route from measured transistor behavior to systematic design-space exploration. The compact model used in this work is tailored for 2D semiconductor transistors and incorporates the key electrostatic, transport, contact, and defect-related mechanisms that govern their current–voltage characteristics [30]. Its circuit-compatible analytical formulation permits rapid and numerically stable evaluation while retaining parameters with direct physical meaning. This combination makes compact modeling especially useful for experimental calibration, sensitivity analysis, and the generation of large, structured device datasets. Compared with self-consistent quantum-transport or technology computer-aided design calculations, compact modeling substantially reduces computational cost and enables the high-throughput simulations needed for machine-learning-assisted device design.

Machine learning (ML) can further extend this capability by learning relationships between device parameters and electrical responses, enabling rapid prediction and optimization across multidimensional design spaces [31]. In semiconductor devices, ML has been applied to surrogate-assisted multiobjective design of 2D FETs [32], simulator acceleration and inverse design of FinFETs [33], and optimization of nanosheet and gate-all-around transistors [34,35]. More broadly, the integration of ML with physical systems extends well beyond electronic-device modeling. Recent advances in intelligent nanophotonics have demonstrated the use of ML for photonic inverse design, optical computing, computational imaging, and intelligent sensing [36], illustrating the increasingly interdisciplinary role of data-driven methods in the design and operation of complex physical systems. Related work in 2D materials and semiconductor devices likewise emphasizes physically meaningful inputs, well-controlled data generation, and integration of physical knowledge into data-driven workflows [37,38,39]. For transistor design, however, low average prediction error alone is insufficient. A surrogate should also preserve physically admissible device behavior, appropriately treat numerical limits, and provide evidence that optimized predictions remain credible within the supported design domain.

An additional distinction is essential between forward prediction and parameter identification. A forward surrogate may accurately predict transfer characteristics from known device parameters without implying that the same electrical curves uniquely determine those parameters in reverse. Different combinations of geometry, mobility, contact resistance, impurity density, and trap density can produce similar electrical responses. Accordingly, inverse recovery must be evaluated independently rather than inferred from forward-model accuracy alone [40]. Likewise, optimization tends to select extreme predictions, where surrogate uncertainty and training-domain support are particularly important. An uncertainty-aware screening strategy is therefore needed before an optimized candidate is interpreted as a meaningful design recommendation [41]. Here, ‘uncertainty-aware’ refers to safeguards against surrogate-induced prediction uncertainty through local training-domain support, cross-model disagreement, validation residuals, and subsequent source-model confirmation; it does not refer to semiconductor lifetime or degradation reliability.

In this work, we develop an uncertainty-aware ML surrogate framework for compact-model-based analysis and design of WSe_2_ *p*-channel FETs. Published experimental output characteristics are first used to calibrate the physics-based S2DS compact model. Once the compact-model parameters are fixed, the same S2DS relation $I_{D}=f\left(V_{{GS}^{’}}V_{{DS}^{’}}\theta \right)$ is used to generate *p*-branch transfer characteristics by sweeping $V_{GS}\;$at two fixed drain biases across a seven-dimensional ensemble spanning channel length, equivalent oxide thickness, hole mobility, contact resistance, impurity density, trap density, and gate-voltage offset. Thus, the experimental output characteristics serve as the compact-model calibration data, whereas the machine-learning surrogate is trained on S2DS-generated transfer characteristics rather than directly on the experimental measurements.

The surrogate explicitly distinguishes resolved current from values limited by the numerical floor and enforces the expected monotonic *p*-channel response within the modeled bias window. From these predicted characteristics, transistor performance is evaluated using on-state current, maximum transconductance, threshold voltage, normalized drain-bias threshold shift, and SS. The multiobjective design stage specifically targets four quantities: maximizing the saturation on-state current, maximizing the saturation-bias maximum transconductance, minimizing the normalized drain-bias threshold shift, and minimizing the deviation of the saturation threshold voltage from a prescribed target. SS is additionally evaluated as a derivative-sensitive measure of *p*-branch curve-prediction fidelity but is not used as a primary optimization objective. The on/off current ratio is not treated as an optimization target because the low-current region is frequently left-censored by the numerical current floor and the present unipolar *p*-channel model does not resolve a complete ambipolar off-state.

Inverse regression and nearest-neighbor analysis are used to assess parameter identifiability separately from forward accuracy, while global Sobol analysis quantifies parameter influence on the electrical responses. Finally, Pareto optimization combined with domain-support, model-disagreement, and residual-based criteria prioritizes candidate designs for direct source-model verification. Because the surrogate learns the response of the underlying S2DS model, the resulting sensitivity, identifiability, and optimization conclusions are conditional on the physical mechanisms and parameter domain represented by the experimentally calibrated source model. Together, these analyses provide a physically grounded framework for forward prediction, performance assessment, and uncertainty-screened multiobjective design within the calibrated compact-model domain.

## 2. Methods

**Figure 1:**
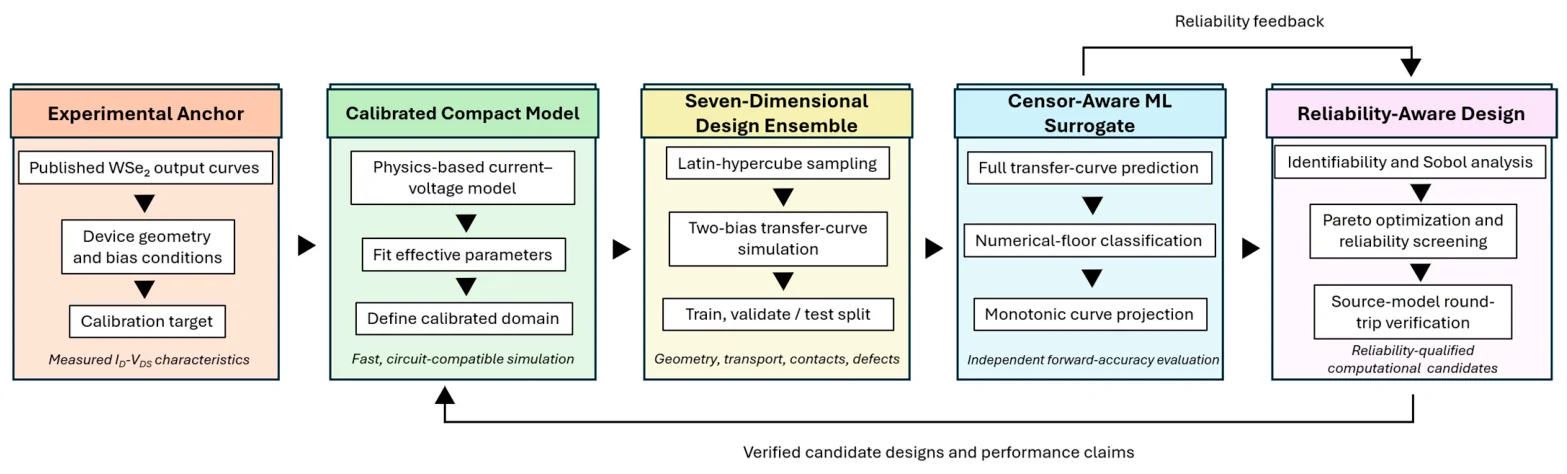
Uncertainty-aware surrogate modeling and compact-model-based design framework for WSe_2_ *p*-channel FETs. Published output characteristics are used to calibrate a physics-based compact model, which generates a seven-dimensional simulation dataset. A censor-aware ML surrogate predicts *p*-branch transfer curves within the modeled bias window and supports identifiability analysis, global sensitivity analysis, Pareto optimization, uncertainty screening, and final source-model verification of selected design candidates.

Figure 1 summarizes the computational workflow used in this study. Published WSe_2_ transistor measurements were first used to establish an experimental anchor for a physics-based compact model [7]. The calibrated model then generated a seven-dimensional device ensemble, which was used to train and independently evaluate a censor-aware ML surrogate. The surrogate subsequently supported identifiability analysis, global sensitivity analysis, multiobjective optimization, uncertainty screening, and final source-model verification of the selected computational candidates.

### 2.1. Experimental Anchor and Compact-Model Calibration

The experimental anchor was the output-characteristic dataset of the monolayer *p*-type WSe_2_ FET reported by Fang et al. [7]. This dataset was selected as a well-established experimental calibration reference rather than as a claim of the current performance limit of WSe_2_ technology. The Fang et al. device provides a well-characterized monolayer *p*-type WSe_2_ FET with documented geometry, contact engineering, and electrical characteristics, and it also serves as a WSe_2_ validation case for the S2DS compact-model framework used here. Its use therefore provides methodological continuity between the experimental reference device, the physics-based source model, and the present ML-assisted workflow. The cleaned dataset contained 211 digitized observations. Calibration used 161 points measured at $V_{GS}=-0.8$, −1.1, −1.4, and −1.7 V, whereas the 50-point curve at $V_{GS}=-0.5\;V$ was reserved for evaluation without refitting. The reported channel length was fixed at $L_{G}=9.4\;\mu m$. The SiO_2_-equivalent oxide thickness was calculated as $EOT=t_{ox}\frac{\varepsilon_{SiO_{2}}}{\varepsilon_{ox}}=\left(17.5\;nm\right)\frac{3.9}{12.5}=5.46\;nm.$

The experimental output characteristics and the subsequently generated transfer characteristics serve different roles in the workflow and should not be interpreted as a point-by-point 1:1 mapping. Both represent different bias slices of the same compact-model response $I_{D}\left(V_{{GS}^{’}}V_{{DS}^{’}}\theta \right)$: output characteristics vary $V_{DS}\;$at fixed $V_{GS}$, whereas transfer characteristics vary $V_{GS}\;$ at fixed $V_{DS}$. The Fang et al. output characteristics are used here to calibrate a single S2DS parameterization; after calibration, that same parameterization is evaluated forward to generate the *p*-branch transfer characteristics used in the ML dataset. Thus, the experimental output data are calibration data for the physics-based source model and are not themselves the ML training labels.

Simulations used a local S2DS OpenVAF/ngspice port [30]. The *p*-channel model used type=−1, a fixed device width of W=1 μm, a source/reference potential of 0 V, and the model parameter $V_{BS}=+40\;V$. Increasingly negative gate voltage increased $|I_{D}|$, and all reported currents were expressed as the positive magnitude $|I_{D}|$. Self-heating, high-field mobility correction and gate-field mobility correction were disabled, SELFHEAT=HIFIELD=GATEFIELD=0, and the overlap-length parameter was set to LOV=0.

Six effective parameters were fitted simultaneously: hole mobility $\mu_{0,h}$(MU0H), contact resistance $R_{c}$, sheet impurity density $N_{imp}$, sheet trap density $D_{trap}$, gate-voltage offset $V_{GS0}$, and output-resistance parameter λ. Calibration used a deterministic custom search consisting of a 64-point Latin-hypercube design with seed 120, together with the manual baseline, followed by three coordinate-refinement rounds with normalized search half-widths of ±0.15, ±0.075, and ±0.035. The complete schedule contained 101 source-model evaluations and did not use a tolerance-based convergence criterion. Fitting was performed in linear current after converting both measured and simulated current magnitudes to μA/μm.

For gate-bias curve g  and measurement point i, the normalized residual was defined as $r_{g,i}=\frac{I_{g,i}^{sim}-I_{g,i}^{meas}}{\underset{i}{max}\left(I_{g,i}^{meas}\right)},$ and the global normalized root-mean-square error was $NRMSE=\sqrt{\frac{1}{N}\sum_{g}\sum_{i}r_{g,i}^{2}}=\sqrt{\frac{1}{N}\sum_{g}\sum_{i}{\left[\frac{I_{g,i}^{sim}-I_{g,i}^{meas}}{\underset{i}{max}\left(I_{g,i}^{meas}\right)}\right]}^{2}},\;$ where N is the total number of calibration observations. The fitted ranges, manual-baseline values, and optimized values were as follows: $\mu_{0,h}$: 100–$400\;cm^{2}\;V^{-1}\;s^{-1}$, baseline $250\;cm^{2}\;V^{-1}\;s^{-1}$, optimized $143.3649\;cm^{2}\;V^{-1}\;s^{-1}$; $R_{c}$: 500–10,000 Ω μm, baseline 5000 Ω μm, optimized 924.1255 Ω μm; $N_{imp}$: $10^{14}$–$10^{17}\;m^{-2}$, baseline $10^{16}\;m^{-2}$, optimized $1.08049\times 10^{16}\;m^{-2}$; $D_{trap}$: $10^{13}$–$10^{16}\;m^{-2}$, baseline $10^{14}\;m^{-2}$, optimized $4.00236\times 10^{13}\;m^{-2}$; $V_{GS0}$: 0–1.2 V, baseline 0.6 V, optimized 0.742633 V; λ: 0–$0.30\;V^{-1}$, baseline $0.20\;V^{-1}$, optimized $0.0465975\;V^{-1}$.

The global NRMSE decreased from 1.30589 for the manual baseline to 0.087142 after calibration. In the leave-one-gate-bias-out analysis, the model was refitted using the remaining three primary calibration curves before prediction of the held-out curve. The resulting NRMSE values were 0.1542, 0.1007, 0.0900, and 0.0443 for $V_{GS}=-0.8$, −1.1, −1.4, and −1.7 V, respectively. By contrast, the separate weak-bias curve at $V_{GS}=-0.5\;V$ was evaluated using the final calibrated parameter set without refitting and produced an NRMSE of 0.6542. The calibration therefore provides a local experimental anchor for the simulated domain but does not establish that the fitted values constitute a unique set of physical material parameters.

In particular, the fitted $D_{trap}$ quantity is treated as an effective sheet-trap coordinate of the S2DS compact model rather than as a conventional experimental extraction of an energy-resolved interface-trap density $D_{it}$. The available DC characteristics do not independently resolve interface-trap physics, and the physical identifiability of $D_{trap}\;$is therefore assessed separately in Section 3.2. Accordingly, its calibrated value should not be interpreted as a uniquely determined experimental trap density.

### 2.2. Seven-Dimensional Design Ensemble and Data Partition

Following calibration, 5000 devices were generated using independent-column Latin-hypercube sampling. The seven varied inputs were $L_{G},\;EOT,\;\mu_{0,h},\;R_{c},\;N_{imp},\;D_{trap},\;and\;V_{GS0}.$ The variables $L_{G}$, EOT, $\mu_{0,h}$, and $V_{GS0}\;$were sampled in linear coordinates, whereas $R_{c}$, $N_{imp}$, and $D_{trap}$ were sampled in ${log}_{10}$ coordinates. The calibrated value of λ and all remaining compact-model settings were held fixed. Pairwise correlations among the sampling coordinates were examined to exclude unintended parameter dependence. The ranges and sampling coordinates for these seven input parameters are summarized in Table 1.

The independent sampling of these seven quantities is a computational design-space assumption used to separate their individual and interaction effects within the compact model; it should not be interpreted as evidence that all seven quantities can be controlled independently during fabrication. In particular, $\mu_{0,h}\;$ and $N_{imp}$ are treated as separate S2DS coordinates in the present ensemble, and no experimentally imposed relation $\mu_{0,h}=f\left(N_{imp}\right)\;$ is assumed. Physical changes in impurity or defect density may simultaneously alter carrier mobility, trap populations, and contact behavior. Similarly, $R_{c}\;$ is treated here as an effective compact-model coordinate. Translation of these independently sampled coordinates into process-level design rules would require experimentally established parameter correlations or a process-aware transport model.

For each sampled device, transfer characteristics were evaluated at $V_{DS}=-0.1$ and −1.0 V over 81 gate-voltage values, $V_{GS,j}=0.5-0.025j,\;j=0,1,\ldots ,80,\;$corresponding to a sweep from +0.5 to −1.5 V. Each device therefore produced 162 current coordinates. The stored response was the total positive current magnitude $|I_{D}|$, in amperes, for W=1 μm. The simulated gate-bias window intentionally covers the *p*-channel branch of the calibrated device. An independent ambipolar electron branch is not represented in the generated dataset. A numerical current floor of $I_{floor}=10^{-12}\;A$ was applied. Values below this boundary were treated as left-censored observations, indicating $|I_{D}|\;\leq I_{floor},$ rather than as exact observations equal to ${log}_{10}\left(I_{floor}\right)=-12$. Accordingly, this numerical floor denotes unresolved source-model current within the modeled *p*-channel window and should not be interpreted as a physically resolved ambipolar off-state current. Failed simulations were retried at most twice using the same parameter vector, without replacement. All 5000 devices completed successfully.

The dataset was partitioned deterministically into 3500 training, 750 validation, and 750 independent test devices. No stratification was applied. Device-level partitioning prevented correlated points from the same transfer curve from appearing in more than one subset. All transformations, scaling parameters, model-selection decisions, probability thresholds, stopping rules, and acceptance criteria were fitted or finalized using only the training and validation data. The independent test outputs remained sealed until the complete modeling workflow was frozen.

### 2.3. Censor-Aware p-Branch Transfer-Curve Surrogate

The surrogate predicted the two *p*-branch transfer curves within the modeled gate-bias window from the seven-dimensional input vector $x={{[L}_{G},\;EOT,\;\mu_{0,h}\;{,log}_{10}\left(R_{c}\right),\;{log}_{10}\left(N_{imp}\right),\;{log}_{10}\left(D_{trap}\right),\;V_{GS0}]}^{T}$. Each input coordinate was standardized using the corresponding training-set mean and standard deviation $z_{k}=\frac{x_{k}-\mu_{k,train}}{\sigma_{k,train}}.$

The regression target was the concatenated 162-coordinate log-current vector $y=\left[\begin{array}{c} {log}_{10}|I_{D}\left(V_{DS}=-0.1\;V\right)|\\ {log}_{10}|I_{D}\left(V_{DS}=-1.0\;V\right)| \end{array}\right].$

Exact coordinates retained their simulated values. Left-censored coordinates were initialized at −12 and iteratively completed in a 48-component full-SVD principal-component subspace. At iteration t, each censored coordinate was updated according to $y_{j}^{\left(t+1\right)}=\min\;\left({\hat{y}}_{j,PCA}^{\left(t\right)},-12\right),j\in C,$ where C denotes the set of censored coordinates. Exact coordinates remained fixed throughout the procedure. Ten fixed completion iterations were performed, with no tolerance-based stopping rule. The retained PCA variance was 0.9999986.

The selected multioutput regressor was an MLP with two hidden layers containing 128 neurons each. The model used rectified-linear-unit activation, Adam optimization, a learning rate of $10^{-3}$, an $L_{2}$ regularization coefficient of $\alpha =10^{-4},$ a batch size of 128, a maximum of 700 iterations, and early stopping with a validation fraction of 0.10 and patience of 35 iterations. Five fixed random seeds, 20,260,731–20,260,735, were used to quantify training variability.

A separate two-output Extra Trees classifier predicted the censored-prefix structure of the two drain-bias sweeps. The classifier used 400 trees, unlimited tree depth, a minimum leaf size of two, all seven input features at each split, and one processing thread. The validation-selected exact-state probability threshold was $p_{exact}\geq 0.60.$ Because the low-current region of a *p*-FET gate sweep must form a contiguous prefix, the classifier outputs were first converted into monotonic pointwise exact-state probabilities. Final curve assembly then followed the fixed sequence: from censor cap to monotonic projection, censor cap, and monotonic projection. This monotonic constraint is applied only to the modeled *p*-channel branch and is not intended to represent a complete ambipolar WSe_2_ transfer characteristic.

Coordinates classified as censored were capped at −12. Each 81-point sweep was subsequently projected using a cumulative maximum in the stored gate-voltage order of ${\tilde{y}}_{j}=\underset{k\leq j}{max}y_{k},$ thereby enforcing the expected monotonic increase in ${log}_{10}|I_{D}|$ as $V_{GS}$ became more negative.

Scalar quantities were extracted from the raw source-model curves using frozen definitions. Endpoint currents were defined as $l_{I,lin}={log}_{10}\left[\frac{\max\left({|I}_{D}(V_{GS}=-1.5\;V,V_{DS}=-0.1\;V)|I_{floor}\right)}{1\;A}\right]$, and $l_{I,sat}={log}_{10}\left[\frac{\max\left({|I}_{D}(V_{GS}=-1.5\;V,V_{DS}=-1.0\;V)|I_{floor}\right)}{1\;A}\right]$. These quantities are reported in decades of amperes, that is, ${log}_{10}\left(A\right)$. At each drain bias, maximum transconductance was calculated from adjacent first differences in the raw current magnitude: $g_{m,j}=|\frac{|I_{D,j+1}|-|I_{D,j}|}{V_{GS,j+1}-V_{GS,j}}|,g_{m,max}=\underset{j}{max}g_{m,j}.$ The surrogate target was ${log}_{10}\left(g_{m,max}/S\right).$ No smoothing was applied before numerical differentiation.

Threshold voltage was obtained using the channel-length-dependent constant-current criterion $I_{ref}=10^{-7}\frac{W/\mu m}{L_{G}/\mu m}\;A.$ The threshold voltage $V_{th}$ was found by linear interpolation of ${log}_{10}\left[\max\left(|I_{D}|,I_{floor}\right)\right]$ as a function of $-V_{GS}$ at the crossing ${log}_{10}|I_{D}|\;={log}_{10}I_{ref}.$ A curve without a valid bracketing interval was assigned an invalid threshold value. A normalized drain-bias threshold shift was calculated from the difference between the constant-current threshold voltages at the two drain biases using $\frac{{\Delta V}_{th}}{{\Delta V}_{DS}}=1000\frac{V_{th,lin}-V_{th,sat}}{|V_{DS,sat}-V_{DS,lin}|}=1000\frac{V_{th,lin}-V_{th,sat}}{0.9}$ and was reported in mV/V. This expression has the conventional normalization commonly used for DIBL; however, because the present design domain contains micrometer-scale channel lengths, the extracted quantity is used here as a drain-bias threshold-shift diagnostic rather than as evidence of classical short-channel drain-induced barrier lowering.

Subthreshold swing was extracted by fitting ${log}_{10}\left[\max\left(|I_{D}|\;,I_{floor}\right)\right]=a+s\left(-V_{GS}\right)$ over resolved current points satisfying $0.001\;I_{ref}\leq \;|I_{D}|\;\leq 0.10I_{ref}.$ The subthreshold swing was then $SS=\frac{1000}{s}mV/dec.$ No smoothing was applied. An SS estimate was considered valid only when at least five eligible points were available, the fitted slope s was positive and finite, and the linear fit achieved $R^{2}\geq 0.98$. These criteria produced 695 valid linear-bias and 693 valid saturation-bias devices in the independent test set. All excluded devices had fewer than five eligible points; none was rejected solely because of the $R^{2}\;$requirement. SS errors were calculated after inverse transformation and are reported directly in mV/dec.

### 2.4. Inverse Identifiability Audit

The inverse models were fitted using the 3500 training devices and evaluated on the 750-device validation set. Each inverse-model input contained the 162-coordinate floor-filled log-current vector and a corresponding 162-coordinate exact/censored mask:$\;q=\left[\begin{array}{c} y_{filled}\\ m_{censor} \end{array}\right]\in R^{324}.$

The outputs were the seven normalized sampling coordinates. For a linearly sampled parameter $x_{k}$, $u_{k}=\frac{x_{k}-x_{k,min}}{x_{k,max}-x_{k,min}},$ whereas for a logarithmically sampled parameter, $u_{k}=\frac{{log}_{10}x_{k}-{log}_{10}x_{k,min}}{{log}_{10}x_{k,max}-{log}_{10}x_{k,min}}.$ A parameter was classified as recoverable only when the selected inverse model achieved validation $R^{2}\geq 0.80$, a median absolute normalized-coordinate error no greater than 0.10, and better performance than the stronger deterministic baseline in both measures.

For the nearest-neighbor ambiguity audit, each independent test device was compared with the training ensemble using Euclidean distance in the frozen, standardized 324-feature curve-plus-mask space: $d_{feature}\left(q,q_{i}\right)={∥z\left(q\right)-z\left(q_{i}\right)∥}_{2}.$

Electrical similarity between the selected pair was reported as $d_{curve}=\sqrt{\frac{1}{162}\sum_{j=1}^{162}{\left(y_{j}-y_{i,j}\right)}^{2}},$ and parameter separation was defined as $d_{param}=\underset{k=1,\ldots ,7}{max}|u_{k}-u_{i,k}|.$ A pair was classified as ambiguous when $d_{curve}\leq 0.05\;\mathrm{decade}$ and $d_{param}\geq 0.20.$

To resolve the collective criterion by input parameter, a parameter-specific ambiguity indicator was additionally defined for each coordinate k. A test–training pair was classified as ambiguous with respect to parameter k when the nearest-training-curve RMSE was no greater than 0.05 decade and the absolute normalized separation in that specific coordinate satisfied $|\Delta {\tilde{\theta }}_{k}|\;\geq 0.20$. The same frozen nearest-neighbor pairs, training-only standardization, and thresholds were used for all seven parameters; no model refitting or threshold adjustment was performed.

### 2.5. Global Sensitivity, Multiobjective Search, Uncertainty Screening, and Source-Model Verification

Global sensitivity analysis used a custom NumPy/SciPy Saltelli-style estimator with scrambled SciPy quasi-Monte Carlo Sobol sampling and seed 20,260,801. For a base sample size N=4096 and D=7  varied parameters, the analysis required $N\left(2+2D\right)=4096\left(2+14\right)=65,536$ surrogate evaluations. First-order, total-effect, and signed second-order indices were computed from the A, B, $AB_{i}$, and $BA_{i}\;$ sample matrices. A total of 128 bootstrap resamples were used to characterize numerical variation.

Multiobjective exploration used a separate scrambled Sobol set containing 262,144 candidates. The minimized objective vector was written as $f\left(x\right)=\left[\begin{array}{c} -l_{I,sat}\\ -l_{g,sat}\\ \Delta V_{th}/\Delta V_{DS}\\ |V_{th,sat}-V_{th,target}| \end{array}\right],$ where $V_{th,target}=0.4982413\;V$ was the median valid $V_{th,sat}\;$among the 3500 training devices. Feasible candidates were required to satisfy $0<\Delta V_{th}/\Delta V_{DS}\leq 6.09219\;mV/V,$ $-0.13190\;V\leq V_{th,sat}\leq 1.04664\;V,$ and to have finite values for all four primary objectives. Candidate $x_{a}$ dominated candidate $x_{b}$ when $f_{j}\left(x_{a}\right)\leq f_{j}\left(x_{b}\right)\mathrm{for}\;\mathrm{all}\;j,$ with strict inequality for at least one objective. The nearest-training distance for candidate x was $d_{NN}\left(x\right)=\underset{i\in T}{min}{∥z\left(x\right)-z\left(x_{i}\right)∥}_{2},$ where T denotes the training set and z is the frozen training-set standardization. The distance criterion was $d_{NN}\leq 1.170277.$

For objective k, model-family disagreement was calculated as the population standard deviation $\sigma_{ens,k}=\sqrt{\frac{1}{M}\sum_{m=1}^{M}{\left({\hat{y}}_{m,k}-{\bar{\hat{y}}}_{k}\right)}^{2}},$ with M=5 model families: linear ridge, quadratic ridge, Extra Trees, histogram-gradient boosting, and MLP. The validation-derived disagreement limits were $\sigma_{ens,I_{on}}\leq 0.071057\;\mathrm{decade},\;\sigma_{ens,g_{m}}\leq 0.047947\;\mathrm{decade},\;\sigma_{ens,\;\Delta V_{th}/\Delta V_{DS}}\leq \frac{0.301249\;mV}{V},$ and $\sigma_{ens,V_{th}}\leq 0.043755\;V.$ Let $q_{0.90,k}$ denote the 90th percentile of the absolute validation residual for objective k. The conservative claims were $l_{I,sat,cons}={\hat{l}}_{I,sat}-q_{0.90,l_{I}},\;l_{g,sat,cons}={\hat{l}}_{g,sat}-q_{0.90,l_{g}},\;\;{\left(\frac{\Delta V_{th}}{\Delta V_{DS}}\right)}_{cons}=\frac{\Delta V_{th}}{\Delta V_{DS}}+q_{0.90,\Delta V_{th}/\Delta V_{DS}}$ and $\delta V_{th,cons}=|V_{th,sat}-V_{th,target}|+q_{0.90,V_{th}}.$ The corresponding residual values were 0.015178 decade, 0.011251 decade, 0.277514 mV/V, and 0.014530 V for $I_{on}$, $g_{m}$, the normalized drain-bias threshold-shift metric, and $V_{th}$, respectively.

A candidate qualified for direct S2DS reevaluation only when it satisfied the nearest-distance criterion and all four disagreement criteria. During the round-trip evaluation, the corresponding objective claim passed when $l_{I,sat,S2DS}\geq l_{I,sat,cons},\;l_{g,sat,S2DS}\geq l_{g,sat,cons},\;{\left(\frac{\Delta V_{th}}{\Delta V_{DS}}\right)}_{S2DS}\leq {\left(\frac{\Delta V_{th}}{\Delta V_{DS}}\right)}_{cons},$ and $|V_{th,sat,S2DS}-V_{th,target}|\leq \delta V_{th,cons}.$

An overall pass required all four conditions to be satisfied. The uncertainty-screening thresholds and conservative claims were frozen before source-model reevaluation. This round trip verifies candidate-specific consistency with S2DS after surrogate prediction, optimization, and post-processing; it does not constitute independent experimental validation or a fabrication-yield guarantee. The uncertainty screen evaluates the support and stability of surrogate predictions; it does not assess semiconductor aging, lifetime, or degradation reliability.

## 3. Results and Discussion

### 3.1. Experimental Anchoring and Independent Surrogate Validation

**Figure 2:**
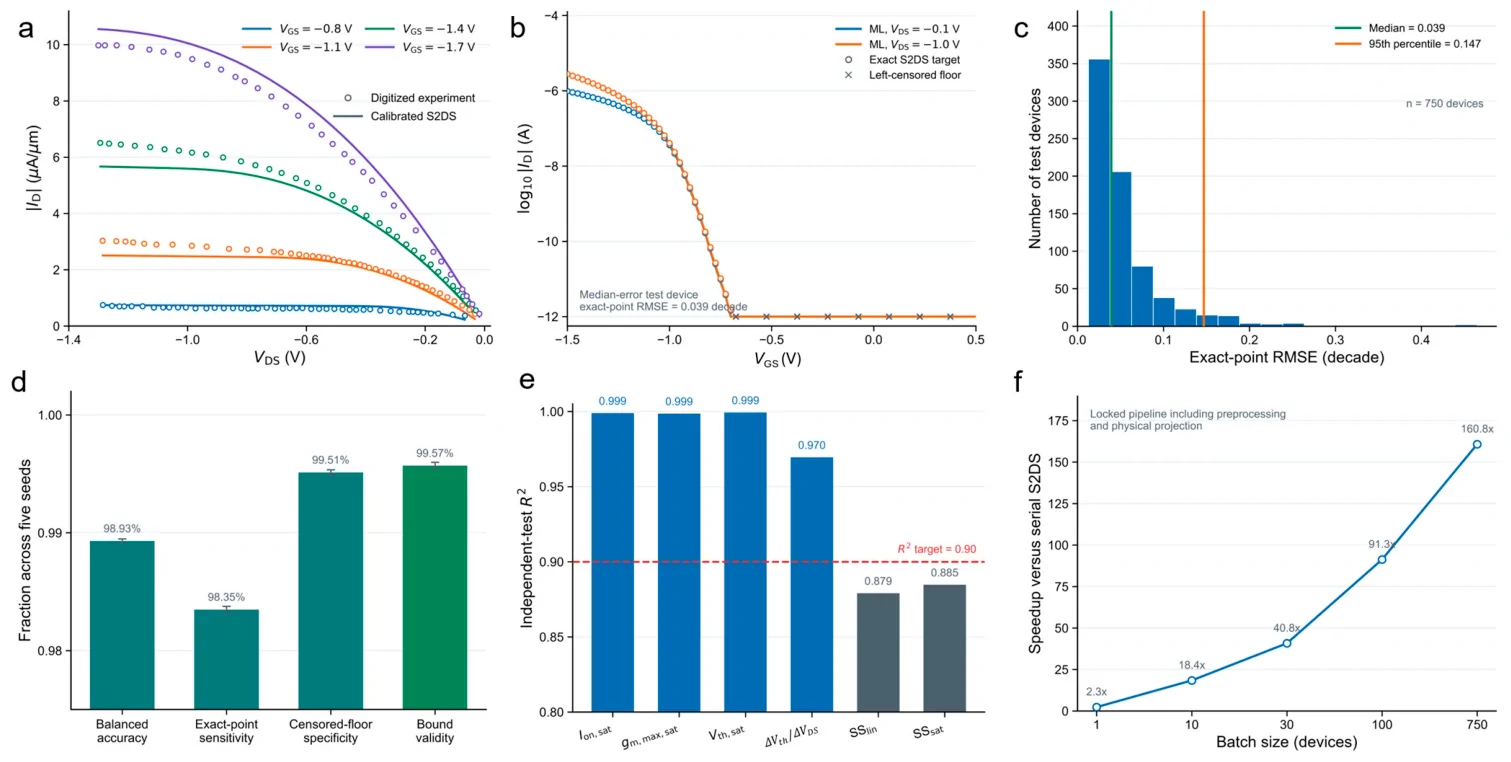
Experimental anchoring and independent surrogate validation. (**a**) Digitized published $I_{D}$-$V_{DS}$ characteristics of a monolayer *p*-type WSe_2_ FET (open symbols) and calibrated compact-model curves (solid lines) at four gate biases. (**b**) Representative median-error independent-test *p*-branch transfer curves at $V_{DS}=-0.1$ and −1.0 V; open circles denote exact current values, crosses denote left-censored floor coordinates, and solid lines denote surrogate predictions. (**c**) Distribution of device-level exact-coordinate RMSE across the 750-device independent test set; vertical lines indicate the median and 95th percentile. (**d**) Five-seed censor-classification and floor-bound-consistency metrics. (**e**) Independent-test $R^{2}$ values for primary scalar outputs and auxiliary subthreshold-swing outputs; the red line marks the prespecified $R^{2}=0.90$ criterion. (**f**) Measured end-to-end surrogate speedup relative to serial source-model simulation as a function of batch size.

Figure 2 distinguishes two complementary levels of model evaluation. First, the physics-based S2DS compact model is anchored to published measurements of a monolayer *p*-type WSe_2_ FET reported by Fang et al. [7]. In Figure 2a, the open symbols represent the digitized experimental output characteristics, whereas the solid lines represent the calibrated S2DS responses. These measurements provide a direct experiment-to-compact-model comparison for the nominal device state. Second, the machine-learning surrogate is evaluated independently against S2DS-generated responses over the seven-dimensional design domain, as shown in Figure 2b–e. The ML model is therefore not trained directly on experimental measurements; rather, it approximates an experimentally anchored physics-based source model. Accordingly, the experimental output characteristics and the S2DS-generated transfer characteristics serve different levels of the validation hierarchy rather than constituting a point-by-point 1:1 comparison. The former constrain the source-model parameterization, whereas the latter define the forward-response space learned by the surrogate.

Calibration used 161 experimental observations at $V_{GS}=-0.8$, −1.1, −1.4, and −1.7 V. Simultaneous adjustment of hole mobility, contact resistance, impurity density, trap density, gate-voltage offset, and the output-resistance parameter reduced the global NRMSE from 1.3059 to 0.08714. The calibrated model reproduces the increase in current with increasingly negative gate bias, the approximately linear low-$|V_{DS}|$ response, and the gradual approach to saturation. A separate weak-bias experimental curve at $V_{GS}=-0.5$ V, which was not used in the final parameter fitting, produced an NRMSE of 0.6542 when evaluated without refitting. The reduced agreement in this weak-current regime indicates that the experimental calibration should be interpreted as a local anchor rather than evidence of uniform predictive accuracy across all bias conditions. Accordingly, the subsequent surrogate-based design conclusions are restricted to the calibrated compact-model domain. The Fang et al. device is used here as a well-established experimental calibration reference, not as a claim of the current state-of-the-art performance limit of WSe_2_ technology. Its selection provides methodological continuity with the S2DS framework, for which the same WSe_2_ device has served as an experimental validation case [30]. Contemporary scaled, wafer-grown, and contact-engineered WSe_2_ devices may occupy substantially different material, geometric, and transport regimes; extending the present framework to those technologies would therefore require a separately calibrated source-model domain.

Figure 2b evaluates full *p*-branch transfer-curve prediction for a sealed-test device at $V_{DS}=-0.1$ and −1.0 V within the modeled gate-bias window. Full-curve learning has previously been demonstrated for FinFET I-V and C-V characteristics using autoencoder-based dimensional reduction [42], and simulator-acceleration studies have shown that data transformations can reduce the severe imbalance between subthreshold and above-threshold current [33]. The present problem contains an additional numerical feature: S2DS clips unresolved current at 10^−12^ A. Those coordinates are not exact current observations but left-censored statements that the latent current is less than or equal to the floor. Treating the floor as an ordinary regression target would create an artificial point mass, overweight agreement with the simulator limit, and distort the learned location of the turn-on boundary. The classifier–regressor construction instead predicts whether a coordinate is resolved and regresses current only where an exact source-model value exists.

The deployed surrogate incorporated a predefined monotonic *p*-FET projection to remove local curve reversals introduced by regression noise. This lightweight, model-agnostic constraint ensured physically consistent curve ordering and current-floor behavior across the full test set within the calibrated bias domain. Unlike physics-integrated architectures that embed analytical current equations and provide broader guarantees for zero-bias behavior, derivative smoothness, and extrapolation [43], the present projection was designed specifically to enforce monotonic consistency during deployment. Accordingly, the 100% monotonic-device rate reflects the behavior of the constrained surrogate rather than the unconstrained MLP alone. This monotonicity constraint applies specifically to the modeled *p*-channel branch and should not be interpreted as a representation of a complete ambipolar WSe_2_ transfer characteristic.

The error distribution in Figure 2c is more informative for subsequent optimization than a single pooled score. Across the 750-device sealed test set, the median exact-coordinate RMSE is 0.039 decade and the 95th percentile is 0.147 decade. Across five independently initialized fits, the exact-coordinate RMSE is 0.07991 ± 0.00277 decade and the mean pointwise *R*^2^ is 0.98909 ± 0.00141. The small seed dependence supports numerical reproducibility, whereas the right-skewed device-level distribution identifies a minority of designs for which error is appreciably larger. This tail is consequential because an optimizer does not sample test devices uniformly; it preferentially selects candidates with unusually favorable predicted objectives, where even a small systematic bias can be amplified.

Figure 2d shows that the numerical-floor task is also well resolved: balanced classification accuracy is 98.93%, exact-coordinate sensitivity is 98.35%, censored-coordinate specificity is 99.51%, and the final floor-bound violation rate is 0.430%. Figure 2e further demonstrates that accurate reconstruction of the transfer curves does not produce equal predictive fidelity for every extracted metric. Saturation-bias $I_{on}$, maximum transconductance, and constant-current threshold voltage achieve $R^{2}$ values of 0.99897, 0.99863, and 0.99935, respectively, while normalized drain-bias threshold shift reaches $R^{2}=0.96954$. The surrogate therefore provides high predictive accuracy for endpoint, threshold-crossing, and transconductance-related quantities. Subthreshold swing provided a stringent assessment of derivative-sensitive prediction, achieving $R^{2}$ values of 0.87910 and 0.88473 at the linear and saturation drain biases, respectively. Because SS is derived from a local logarithmic slope, it represents a more demanding prediction target than endpoint- or threshold-based metrics. The resulting performance demonstrates that the surrogate remains effective even for locally extracted quantities and supports its use as a diagnostic measure of *p*-branch curve fidelity. The complete independent-test results are summarized in Table 2.

Figure 2f places the computational benefit in the correct context. The measured end-to-end speedup grows from 2.32× for one device to 160.77× for 750 devices, including preprocessing, censor classification, curve projection, and scalar extraction. Reported accelerations relative to TCAD or NEGF can be orders of magnitude larger [33] because those source calculations solve substantially more expensive field or quantum-transport problems. S2DS is already an efficient compact model, so isolated-device acceleration is not the principal contribution. The practical gain appears at the workflow level, where 65,536 evaluations are required for Sobol analysis and 262,144 candidates are screened during optimization.

### 3.2. Accurate Forward Prediction Does Not Imply Unique Parameter Recovery

Strong forward prediction does not establish that the seven device inputs can be uniquely recovered from two transfer curves. Let the source model be written as y = f(θ), where θcontains *L*_G_, EOT, mobility, *R*_c_, *N*_imp_, *D*_trap_, and *V*_GS0_, and y contains the two sampled transfer curves. Forward accuracy tests whether f(θ) can be approximated. Parameter extraction requires the inverse relation to be sufficiently one-to-one. Locally, this stronger condition depends on whether perturbations of different parameters produce distinguishable directions in curve space; if columns of the response Jacobian are weak or nearly collinear, the inverse problem is ill-conditioned even when the forward model is highly accurate.

**Figure 3:**
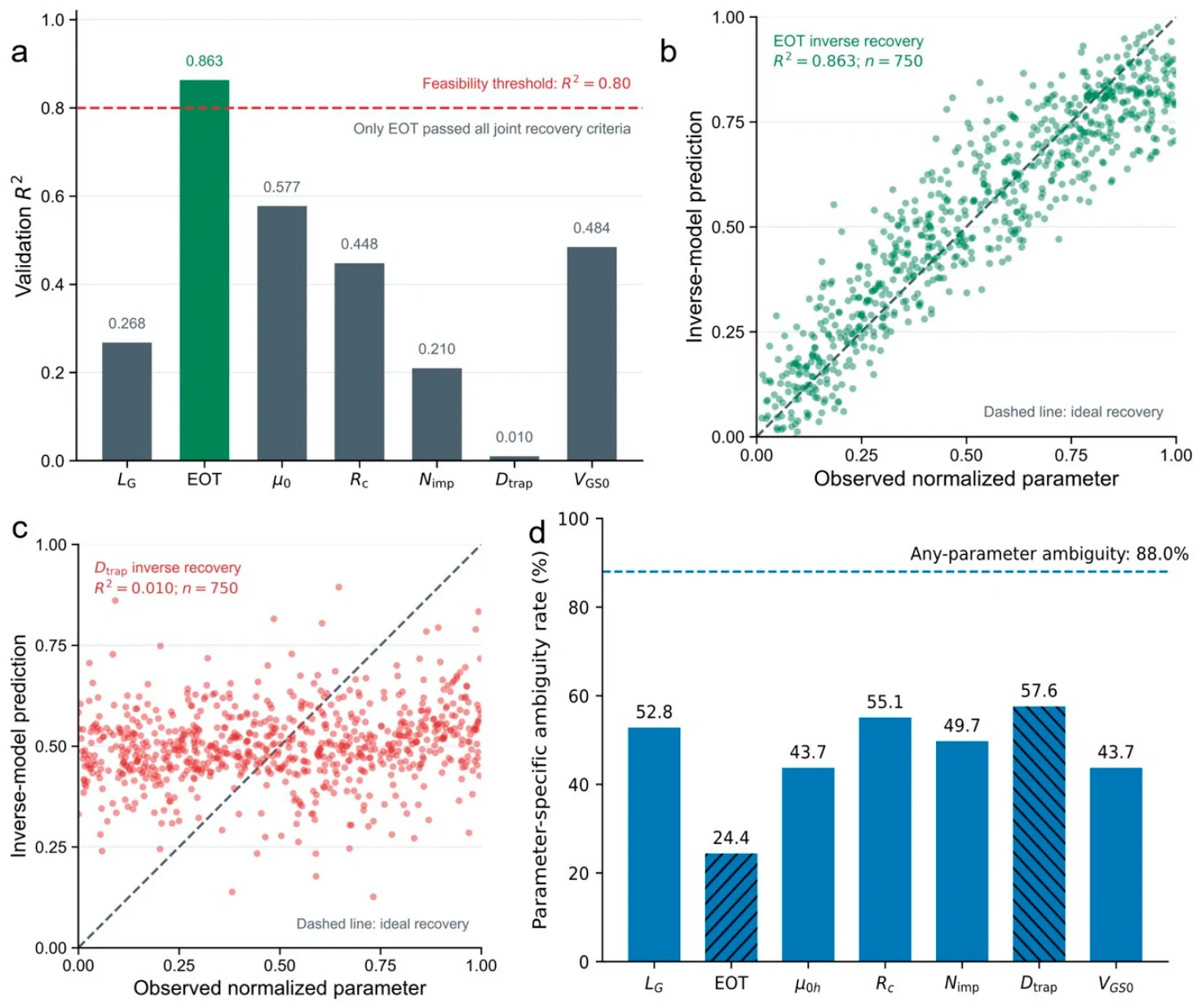
Identifiability audit of the seven-variable inverse problem. (**a**) Validation $R^{2}$ values of selected inverse regressors for the seven varied inputs; only EOT satisfies the prespecified joint recovery criteria. (**b**) Observed versus predicted normalized EOT values, illustrating the recoverable case. (**c**) Observed versus predicted normalized trap-density values, illustrating regression toward the center of the design range when the electrical curves provide insufficient information. (**d**) Parameter-specific nearest-neighbor ambiguity rates for the 750-device sealed independent test set. For each parameter, ambiguity requires a nearest-training-curve RMSE no greater than 0.05 decade and an absolute normalized difference of at least 0.20 in that specific input; the dashed reference indicates the collective any-parameter ambiguity rate.

Figure 3a shows that EOT exhibits the strongest and most distinct electrical signature, achieving an inverse $R^{2}$ of 0.86294 and satisfying the prespecified recovery criteria of $R^{2}\geq 0.80$ and median absolute normalized error no greater than 0.10. The remaining values are 0.57709 for mobility, 0.48424 for *V*_GS0_, 0.44799 for *R*_c_, 0.26776 for *L*_G_, 0.20984 for *N*_imp_, and 0.01018 for *D*_trap_. The particularly low recovery accuracy for $D_{trap}$ is important for interpreting the calibrated model. In the present workflow, $D_{trap}$ is an effective S2DS compact-model coordinate rather than a uniquely extracted experimental interface-trap density. Its inverse $R^{2}\;$of 0.01018 indicates that the available pair of *p*-branch transfer curves contains insufficient independent information to recover this coordinate uniquely over the sampled domain. The inverse audit revealed substantial differences in the recoverability of the seven compact-model parameters. EOT exhibited the strongest and most distinct electrical signature and was the only parameter satisfying the prespecified joint recovery criteria. The remaining parameters exhibited varying degrees of response equivalence, demonstrating that the inverse audit can distinguish uniquely observable quantities from parameters that support multiple electrically equivalent design solutions. This finding does not affect the accuracy of the forward surrogate; rather, it clarifies the level of parameter interpretation supported by the available electrical observables. The contrast between EOT and $D_{trap}$ in Figure 3b,c is consistent with their modeled electrical roles. EOT directly controls the gate capacitance and therefore alters the gate-voltage position and width of the turn-on transition across much of the transfer curve. Its effect is broad and comparatively difficult to reproduce through variation in a single alternative parameter. By contrast, the electrical signature associated with the effective $D_{trap}$ coordinate is weak and can be compensated by changes in other electrostatic and transport coordinates, including EOT, $V_{GS0}$, and $N_{imp}$, under the selected steady-state bias conditions. The clustering of inverse predictions near the center of the sampled range therefore reflects response-equivalent compact-model parameter combinations rather than a uniquely recoverable physical trap density.

The nearest-neighbor audit provides architecture-independent evidence of extensive response equivalence within the design space. Using the sealed 750-device independent test set, 660 devices (88.00%) have a training-set neighbor with a curve RMSE no greater than 0.05 decade while differing by at least 0.20 in one or more normalized input coordinates. The parameter-resolved analysis in Figure 3d shows that the ambiguity is not attributable to a single model coordinate. The corresponding ambiguity rates are 52.80% for $L_{G}$, 24.40% for EOT, 43.73% for $\mu_{0,h}$, 55.07% for $R_{c}$, 49.73% for $N_{imp}$, 57.60% for $D_{trap}$, and 43.73% for $V_{GS0}$. The corresponding parameter-specific nearest-neighbor distributions are provided in Supplementary Figure S1a–g. EOT exhibits the lowest ambiguity rate, consistent with its comparatively strong inverse recoverability, whereas $D_{trap}$ exhibits the highest ambiguity rate. Importantly, substantial ambiguity is also observed for $L_{G}$, $R_{c}$, $N_{imp}$, mobility, and $V_{GS0}$, demonstrating that the collective ambiguity is not governed solely by trap density or contact resistance. The independently evaluated sealed-test ambiguity rates are strongly anticorrelated with the validation-set inverse-model $R^{2}\;$values (Spearman ρ=−0.847), providing consistent evidence from two complementary identifiability diagnostics. These results show that materially separated compact-model parameter vectors can produce closely matched electrical responses, thereby supporting set-valued rather than uniquely determined inverse design.

This distinction clarifies how the present result relates to previous inverse-design and parameter-fitting work. Kim et al. explicitly recognized that semiconductor inverse design is non-injective and evaluated whether a predicted design reproduces the target specifications rather than whether it reconstructs the original geometry [33]. That is a set-valued design problem. Bennett et al. demonstrated that a neural network can return TCAD parameters that reproduce measured WS_2_ transfer curves with high *R*^2^ and can scale to a larger compact-model parameter set [44]. Such results establish efficient fitting and the existence of a response-equivalent parameter set. They do not, by curve agreement alone, prove that the returned set is the unique physical parameterization. The present audit makes that evidentiary distinction explicit by testing inverse recovery and curve-space ambiguity separately. The surrogate is therefore particularly well suited to forward evaluation and set-valued inverse design. It can identify multiple parameter combinations expected to achieve a desired electrical response, providing flexibility in the selection of feasible design solutions.

### 3.3. Global Sensitivity Reveals Output-Specific and Bias-Dependent Control

Variance-based Sobol analysis [45,46] was used to determine how uncertainty in the seven inputs propagates to scalar metrics and to the transfer curves over the frozen compact-model domain. First-order indices measure the contribution of one parameter acting alone, whereas total-effect indices include all interactions involving that parameter. These indices are therefore conditional on the sampled ranges, the assumption that the inputs are varied independently, the two selected drain biases, and the fixed extraction definitions. Accordingly, the resulting sensitivity rankings characterize the declared factorized compact-model domain and should not be interpreted as universal material-property rankings or as evidence that the corresponding quantities are independently tunable in fabrication.

**Figure 4:**
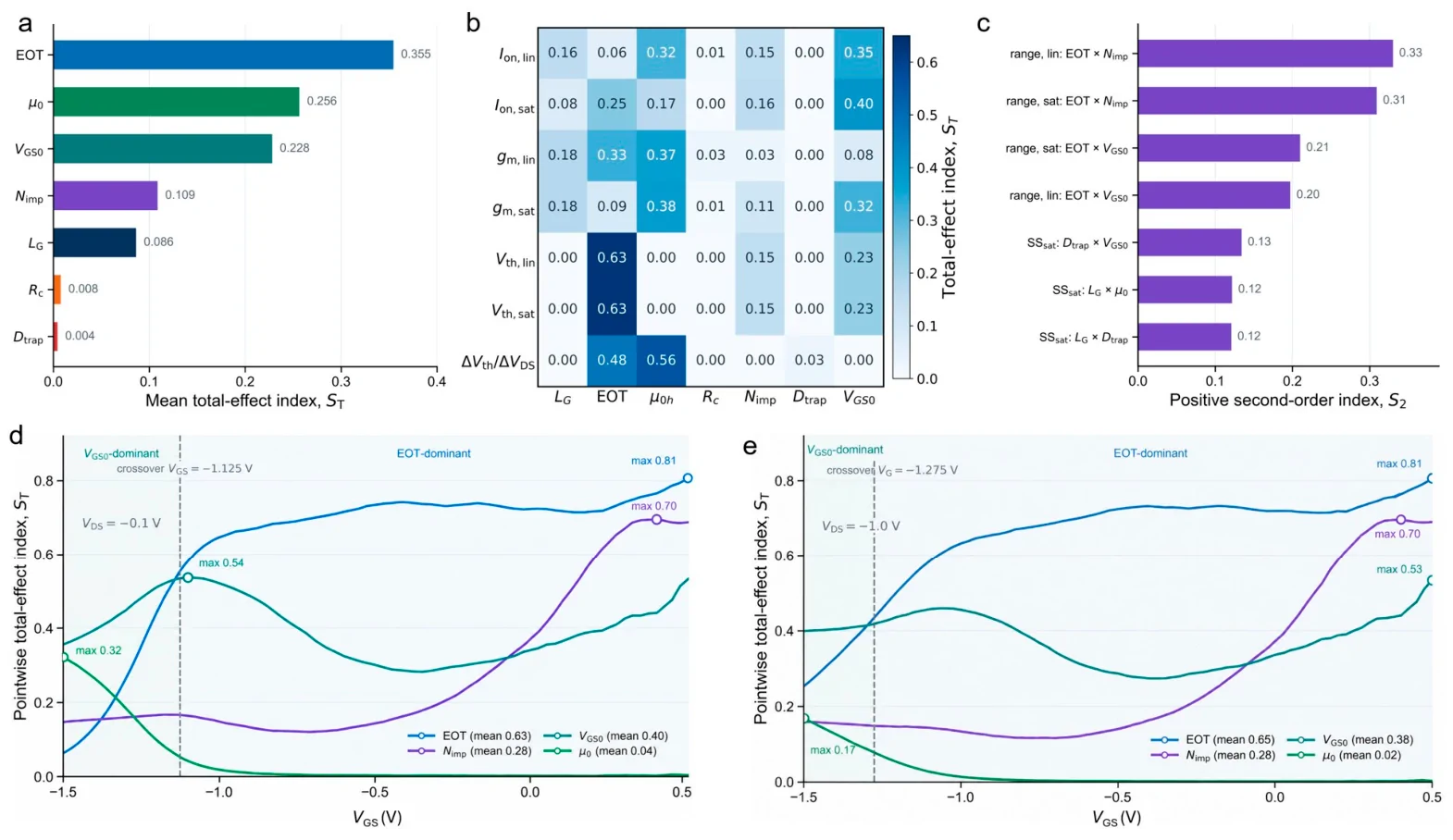
Variance-based global sensitivity analysis within the frozen seven-dimensional compact-model domain. (**a**) Mean total-effect Sobol ranking averaged over the seven scalar outputs considered in the sensitivity analysis. (**b**) Target-resolved total-effect indices for the scalar outputs. (**c**) Largest positive second-order Sobol interactions across scalar and transfer-curve-summary outputs. (**d**) Pointwise total-effect sensitivity profiles along the transfer curve at $V_{DS}=-0.1\;V$. (**e**) Corresponding pointwise profiles at $V_{DS}=-1.0\;V$, showing the bias-dependent contributions of EOT, mobility, impurity density, and gate-voltage offset. In (**d**,**e**), the vertical dashed line marks the crossover $V_{GS}\;$at which the pointwise total-effect indices of EOT and $V_{GS0}\;$are equal, separating the $V_{GS0}$-dominant and EOT-dominant regimes.

Averaged over the seven scalar outputs considered in the sensitivity analysis, Figure 4a ranks EOT first with mean total-effect index S_T_ = 0.3545, followed by mobility (0.2565), *V*_GS0_ (0.2281), *N*_imp_ (0.1086), and *L*_G_ (0.0862). $R_{c}$ and $D_{trap}$ have much smaller mean total effects of 0.00763 and 0.00430, respectively, within the present sampled domain. The combination of low $D_{trap}\;$sensitivity and very poor inverse recoverability supports treating $D_{trap}\;$as a weakly expressed effective compact-model coordinate under the selected DC observables rather than as a uniquely determined experimental interface-trap density. Sensitivity and identifiability nevertheless remain distinct concepts. A parameter may strongly change an output yet remain unrecoverable when another parameter produces a similar change. Mobility is the clearest example: it has a substantial forward influence, but its current-scaling effect is partially confounded with *L*_G_, *V*_GS0_, EOT, and *N*_imp_.

The target-resolved indices in Figure 4b reveal the underlying physical partitioning more clearly. Saturation-bias *I*_on_ is controlled most strongly by *V*_GS0_ (S_T_ = 0.3953), followed by EOT, mobility, and *N*_imp_. *V*_GS0_ translates the effective gate overdrive, EOT controls electrostatic coupling, mobility scales channel transport, and *N*_imp_ modifies the electrostatic charge environment. Because $\mu_{0,h}\;$and $N_{imp}\;$were sampled independently in the present ensemble, these Sobol indices separate their compact-model response contributions and do not imply that impurity engineering can change $N_{imp}\;$without simultaneously affecting mobility or other material properties in a physical device. Linear-regime *I*_on_ is shared among *V*_GS0_, mobility, *L*_G_, and *N*_imp_, consistent with a regime in which both charge control and channel conductance matter. Mobility dominates *g*_m,max_ at both drain biases (S_T_ = 0.3669 and 0.3760), which is consistent with transconductance measuring the rate at which gate-induced charge modulation is converted into drain current. In contrast, *V*_th_ is controlled primarily by EOT (S_T_ = 0.6297 and 0.6320), with secondary contributions from *V*_GS0_ and *N*_imp_, because all three parameters shift the gate-voltage location of the turn-on transition.

The normalized drain-bias threshold-shift metric is influenced primarily by mobility and EOT in the present analysis, with total effects of 0.5571 and 0.48, respectively. This ranking should be interpreted through the extraction procedure rather than as a statement about classical short-channel drain-induced barrier lowering. The simulated devices have micrometer-scale channel lengths, and the metric is obtained from the difference between two constant-current threshold estimates normalized by the drain-bias difference. Any parameter that changes the transfer-curve slope or shifts the constant-current crossing differently at the two drain biases can therefore contribute to its variance. The same crossing sensitivity helps explain why this metric is more difficult to predict than $I_{on}$ or $V_{th}$, while SS, which depends on a local logarithmic slope, remains the most demanding scalar quantity.

Figure 4c further shows that the dominant behavior is not purely additive. The largest positive second-order interaction is EOT × *N*_imp_ for the linear-bias dynamic range (S_2_ = 0.330), with a corresponding value of about 0.31 at saturation bias. EOT × *V*_GS0_ contributes approximately 0.20–0.21. These interactions are consistent with a coupled electrostatic interpretation: EOT sets the strength of gate coupling, while impurity charge and gate-voltage offset shift the effective potential required to enter the resolved-current regime. Changing either electrostatic shift therefore changes the apparent influence of EOT. Additional interactions involving *D*_trap_ × *V*_GS0_, *L*_G_ × mobility, and *L*_G_ × *D*_trap_ occur mainly for SS-related outputs. Because SS did not meet the independent-test criterion, those values are best treated as model-mechanism hypotheses rather than primary design conclusions.

The pointwise profiles in Figure 4d,e show that sensitivity also changes along a single transfer curve. Mobility is most influential in the strongly conducting region, where it scales current magnitude. EOT, *N*_imp_, and *V*_GS0_ become more important near the transition between resolved and floor-limited current because they determine where that transition occurs and how rapidly the resolvable window opens. In the censored region, the reported sensitivity describes movement of the predicted censoring boundary and the latent completed curve.

Taken together, the Sobol and identifiability results provide complementary guidance within the declared compact-model domain. EOT is both influential and comparatively observable; mobility is influential but confounded; $V_{GS0}\;$ and $N_{imp}$ control electrostatic placement through direct and interaction effects; and $R_{c}\;$ and $D_{trap}\;$ are weakly expressed under the present long-channel bias conditions and sampled ranges. In particular, the low mean Sobol index of $R_{c}\;$should not be generalized to scaled WSe_2_ FETs. As channel length decreases and channel resistance is reduced, the relative contribution of contact resistance can become substantially more important, especially when contact injection and electrostatic screening differ from those represented by the present effective contact model. Thus, the current $R_{c}$ ranking is a domain-specific result rather than a statement that contacts are intrinsically unimportant in WSe_2_ transistors. This classification is therefore most useful for distinguishing parameter influence and observability within the present model domain, rather than as a universal ranking of fabrication priorities.

### 3.4. Multiobjective Optimization and Uncertainty Screening

Machine-learning-assisted Pareto optimization has previously been applied to 2D FETs using quantum-transport simulations [32] and to advanced GAAFETs using calibrated TCAD surrogates [35]. Those studies established the value of nondominated search for exposing tradeoffs that cannot be represented by one weighted objective. The present contribution extends Pareto optimization by combining nondominated search with local training-domain support, cross-model consistency, validation-residual safeguards, and direct source-model confirmation.

The four-objective search evaluated 262,144 Sobol candidates. After physical and learned-domain guards, 222,351 remained feasible, and nondominated sorting produced a 48-design Pareto set. The reduction reflects genuine competition among saturation-bias *I*_on_, _gm,max_, the normalized drain-bias threshold-shift metric, and proximity to the threshold-voltage target. As in prior 2D-FET optimization, improved gate control or stronger conduction does not move every objective in the same favorable direction [32]. Here, larger *I*_on_ or *g*_m,max_ can be accompanied by a larger normalized drain-bias threshold-shift metric, a larger threshold deviation, or weaker support from the training ensemble. The resulting Pareto set is therefore a family of application-dependent compromises rather than a unique optimum.

The highest-ranked uncertainty-qualified candidate, P5_049995, has $L_{G}=5.2138\;\mu m$, EOT =3.1326 nm, $\mu_{0,h}=204.30\;cm^{2}\;V^{-1}\;s^{-1}$, $R_{c}=1811.98\;\Omega \cdot \mu m$, $N_{imp}=1.9885\times 10^{16}\;m^{-2}$, $D_{trap}=3.3754\times 10^{13}\;m^{-2}$, and $V_{GS0}=0.55499$ V. Its conservative objective values are ${log}_{10}\left(I_{on,sat}\right)=-4.6316$, ${log}_{10}\left(g_{m,max,sat}\right)=-4.2620$, drain-bias threshold shift =2.6990 mV/V, and an absolute threshold-target deviation of 0.03777 V. More broadly, the nondominated candidates identify favorable tradeoffs among electrostatic control, transport, contact effects, and threshold placement within the sampled compact-model domain. These parameter vectors should be interpreted as source-model design hypotheses rather than directly realizable fabrication recipes, because several inputs are effective model coordinates and are not necessarily independently controllable in physical devices.

**Figure 5:**
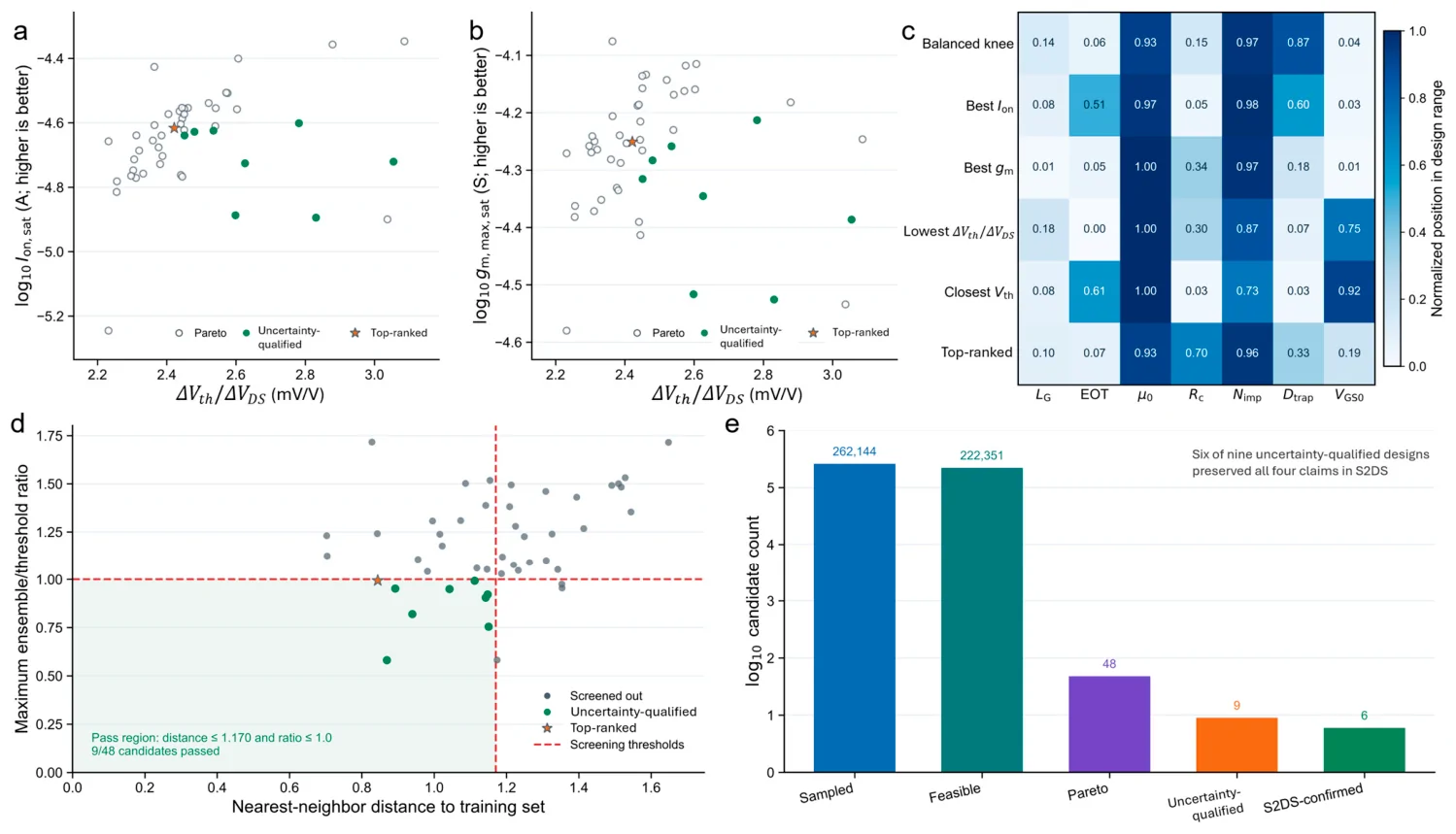
Multiobjective design, uncertainty screening, and candidate attrition within the sampled compact-model domain. (**a**) Saturation on-state current, $I_{on,sat}$, versus the normalized drain-bias threshold-shift metric, $\Delta V_{th}/\Delta V_{DS}$, for the 48-member Pareto set. (**b**) Corresponding projection of saturation-bias maximum transconductance, $g_{m,\max,sat}$, versus $\Delta V_{th}/\Delta V_{DS}$. Open circles denote Pareto candidates, filled green circles denote uncertainty-qualified candidates, and the star identifies the top-ranked candidate. (**c**) Normalized input coordinates for representative Pareto solutions selected by different objective roles, including the balanced knee, best $I_{on}$, best $g_{m}$, lowest $\Delta V_{th}/\Delta V_{DS}$, closest $V_{th}$, and top-ranked candidate. (**d**) Uncertainty screen based on nearest-training-set distance and the maximum model-ensemble-to-threshold ratio; candidates satisfying both frozen criteria are retained for source-model reevaluation. (**e**) Attrition of the 262,144 sampled candidates through feasibility filtering, Pareto selection, uncertainty qualification, and final S2DS confirmation. Nine Pareto candidates satisfied the uncertainty-screening criteria, and six retained all four conservative objective claims after direct S2DS reevaluation.

The input heat map in Figure 5c further illustrates how different parameter combinations can produce competitive objective values within the sampled compact-model domain. High mobility and high $N_{imp}\;$ recur among several representative Pareto candidates, whereas EOT, $R_{c}$, $D_{trap}$, and $V_{GS0}\;$ vary more substantially. This pattern reflects compensation among independently sampled model coordinates rather than a universal conclusion that increasing impurity density independently improves device performance. Because $\mu_{0,h}\;$ and $N_{imp}\;$ were varied independently in the computational ensemble, the optimizer can combine favorable transport with electrostatic shifts associated with $N_{imp}$, EOT, or $V_{GS0}$. In physical WSe_2_ devices, however, impurity density, mobility, trap populations, and contact properties can be correlated. The parameter combinations in Figure 5c should therefore be interpreted as compact-model design hypotheses for subsequent higher-fidelity or experimental evaluation, not as prescriptions to independently tune these quantities during fabrication.

Figure 5d applies three complementary safeguards against selection-induced surrogate error. The nearest-training distance tests whether a candidate lies in a region supported by the simulated ensemble; it is a geometric extrapolation check. Disagreement among ridge, quadratic-ridge, Extra Trees, histogram-gradient-boosting, and MLP models tests whether the favorable prediction is stable across model classes; it is a model-form uncertainty check. Finally, the 90th-percentile absolute validation residual is applied against each objective to penalize finite predictive error. They address different numerical failure modes and are intentionally conservative when used together.

The uncertainty-screening framework prioritized nine Pareto candidates with strong local training-domain support and stable predictions across multiple model families. Following conservative residual adjustment and direct S2DS reevaluation, six designs retained all four objective claims. Prior multiobjective-optimization studies have emphasized cross-checking optimized points with the originating physics model because generic ML surrogates do not strictly obey physical laws [32], and GAAFET cross-validation has shown larger deviations for RF objectives derived from $g_{m}\;$ and $r_{o}\;$than for direct current-based objectives [35]. The resulting attrition funnel therefore quantifies the predictive support and source-model consistency of optimized candidates; it should not be interpreted as semiconductor lifetime reliability, fabrication yield, or long-term device stability.

### 3.5. Source-Model Round-Trip Evaluation

All nine uncertainty-qualified candidates were reevaluated with the source S2DS model. This step provides candidate-level confirmation after large-scale surrogate-assisted optimization and directly evaluates whether favorable predicted outcomes are preserved by the source model. Candidate-specific reevaluation therefore complements population-level test accuracy by directly assessing the optimized region of the compact-model design space.

Direct S2DS reevaluation confirmed that six prioritized designs retained all four conservative performance claims. At the individual-target level, the normalized drain-bias threshold-shift claim passes for 9/9 candidates, $I_{on,sat}\;$ for 8/9, $g_{m,\max,sat}$ for 8/9, and $V_{th,sat}\;$for 7/9. The mean absolute surrogate-to-S2DS differences are 0.11405 mV/V for the drain-bias threshold-shift metric, 0.00923 decade for *I*_on,sat_, 0.00957 decade for *g*_m,max,sat_, and 0.01162 V for *V*_th,sat_; the corresponding maximum errors are 0.26078 mV/V, 0.03351 decade, 0.03123 decade, and 0.01628 V. The small surrogate-to-S2DS differences demonstrate strong candidate-level consistency, including for designs located near stringent acceptance boundaries. The complete round-trip error statistics and source-model claim outcomes are provided in Table 3.

**Figure 6:**
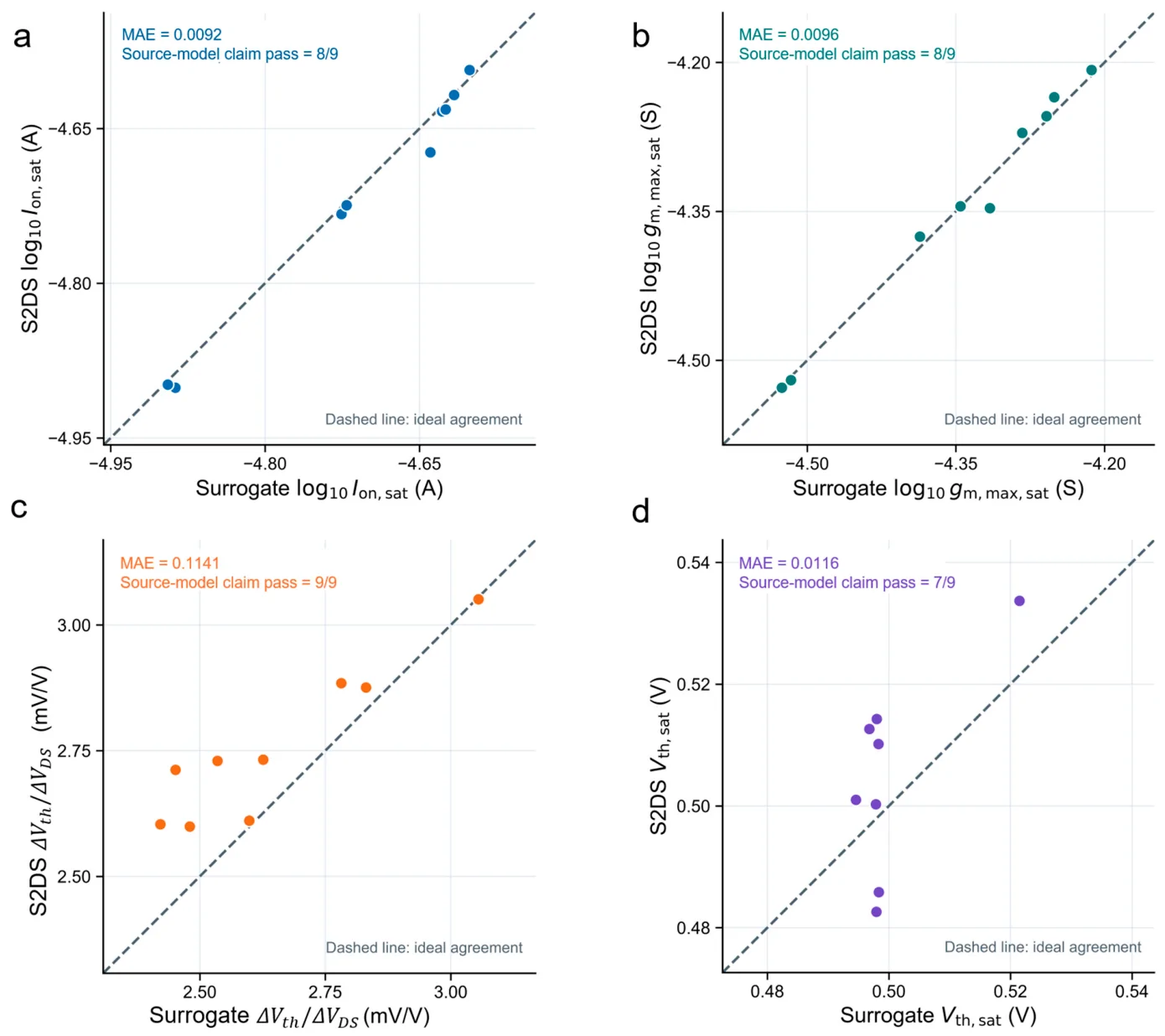
Source-model round-trip confirmation of the nine uncertainty-qualified computational designs. Surrogate predictions are compared with newly executed S2DS results for (**a**) saturation on-state current, $I_{on,sat}$; (**b**) saturation-bias maximum transconductance, $g_{m,max,sat}$; (**c**) normalized drain-bias threshold shift, $\Delta V_{th}/\Delta V_{DS}$; and (**d**) saturation threshold voltage, $V_{th,sat}$. The dashed diagonal line in each panel indicates ideal agreement between the surrogate prediction and the S2DS result. Six candidates preserve all four conservative source-model claims.

Threshold-voltage confirmation remained strong at 7/9 candidates under a particularly narrow target-deviation criterion, complementing its high independent-test $R^{2}$ of 0.99935. The candidate-level comparisons between the surrogate predictions and the newly executed S2DS results for all four objectives are shown in Figure 6. This result highlights the value of candidate-level verification for resolving small local deviations that are not fully represented by global accuracy metrics. This distinction parallels the larger errors previously observed for derivative-based RF objectives during TCAD cross-validation [35] and the difficulty of reproducing SS from predicted curves [33]: the stability of a final design claim depends on the extraction functional and the acceptance margin, not only on curve-level *R*^2^. The six surviving candidates, including P5_049995, P5_184395, P5_098211, P5_058351, P5_049131, and P5_039461, are therefore source-model-confirmed computational candidates under the frozen sampling, extraction, and screening procedures. The round trip verifies consistency with S2DS after optimization.

The present round-trip analysis verifies the optimized candidates against the originating S2DS source model but does not constitute independent experimental validation of the ML-guided designs. Such validation would require fabrication and measurement of WSe_2_ FETs spanning selected uncertainty-qualified parameter regimes, followed by transfer-characteristic measurements under bias conditions consistent with the present simulations. The experimentally measured characteristics could then be evaluated using the same definitions of $I_{on,sat}$, $g_{m,\max,sat}$, the normalized drain-bias threshold-shift metric, and $V_{th,sat}\;$ used in the multiobjective analysis. Alternatively, contemporary published WSe_2_ datasets with sufficiently documented device geometry, dielectric properties, contact characteristics, material state, and measurement conditions could provide an external basis for recalibration and validation. Such studies would be an important next step for assessing whether the present uncertainty-aware workflow transfers to contemporary wafer-scale, defect-engineered, synthetic-material, scaled-channel, or differently contact-engineered WSe_2_ technologies represented in the recent literature and studies [47,48,49,50,51,52,53], beyond the current experimentally calibrated compact-model domain.

The present framework is therefore intentionally scoped to the experimentally calibrated *p*-channel S2DS domain examined here. The seven compact-model coordinates are sampled independently for response-surface interrogation and should not be interpreted as independently controllable fabrication variables; physical correlations among mobility, impurity density, trap populations, and contact resistance are not imposed. Likewise, the modeled transfer characteristics represent the *p*-channel branch within the selected bias window and do not include a separately calibrated ambipolar electron branch. Finally, the micrometer-scale channel-length range and effective contact representation limit extrapolation of the present sensitivity and optimization results to aggressively scaled WSe_2_ transistors, where contact resistance, electrostatic screening, and short-channel effects may become substantially more important. Extending the workflow to contemporary wafer-scale or scaled-channel WSe_2_ technologies will therefore require technology-specific experimental calibration and regeneration of the corresponding compact-model design domain.

## 4. Conclusions

This work presents an uncertainty-aware machine-learning framework for compact-model-based analysis and multiobjective design of WSe_2_ *p*-channel FETs. Using an experimentally calibrated S2DS compact model, the surrogate accurately reproduced *p*-branch transfer characteristics across a seven-dimensional design space while preserving the numerical current-floor boundary and the expected monotonic *p*-channel response within the modeled bias window. Strong independent-test performance for both curve prediction and extracted scalar metrics enabled efficient large-scale design exploration.

The identifiability analysis showed that accurate forward prediction does not imply unique parameter recovery. EOT exhibited the strongest inverse recoverability, whereas several other compact-model coordinates showed substantial response equivalence, with $D_{trap}\;$particularly weakly identifiable. The parameter-specific nearest-neighbor audit further confirmed that ambiguity is distributed across multiple inputs rather than being attributable to a single parameter. Global Sobol analysis quantified the output-specific and bias-dependent influence of geometry, electrostatics, transport, contacts, impurity density, trap density, and gate-voltage offset within the declared compact-model domain.

Multiobjective optimization identified Pareto tradeoffs among saturation on-state current, saturation-bias maximum transconductance, normalized drain-bias threshold shift, and threshold-voltage placement. Uncertainty screening based on local training-domain support, cross-model disagreement, and validation-residual safeguards prioritized nine candidates for direct S2DS reevaluation, of which six retained all four conservative source-model claims. These results demonstrate that surrogate-assisted design can be made more credible by separating forward accuracy from inverse identifiability and by requiring candidate-level confirmation against the originating physics-based model.

The resulting design recommendations remain conditional on the experimentally calibrated S2DS domain and should be interpreted as compact-model design hypotheses rather than direct fabrication prescriptions. Future extensions should incorporate technology-specific calibration to contemporary wafer-scale, scaled-channel, and ambipolar WSe_2_ devices, together with experimentally supported correlations among transport, impurity, trap, and contact parameters. Overall, the proposed workflow provides an efficient and interpretable strategy for uncertainty-aware WSe_2_ transistor design while maintaining explicit boundaries on the physical claims supported by the source model.

## Figures and Tables

**Table 1:** Seven-dimensional compact-model design domain.

Input	Minimum	Maximum	Sampling Coordinate
$L_{G}$ (μm)	4.70	10.00	Linear
EOT (nm)	2.73	8.19	Linear
$\mu_{0,h}$ ($cm^{2}\;V^{-1}\;s^{-1}$)	71.68	215.05	Linear
$R_{c}$ (Ω μm)	200.00	4620.63	Logarithmic
$N_{imp}$ ($m^{-2}$)	$2.16\times 10^{15}$	$2.16\times 10^{16}$	Logarithmic
$D_{trap}$ ($m^{-2}$)	$1.00\times 10^{13}$	$4.00\times 10^{14}$	Logarithmic
$V_{GS0}$ (V)	0.4426	1.0426	Linear

**Table 2:** Sealed 750-device test performance.

Output	*R* ^2^	RMSE	Valid Devices
*p*-branch curve, exact coordinates	0.9891 mean pointwise	0.0799 decade	750
log_10_ *I*_on,sat_	0.99897	0.01051 decade	750
log_10_ *g*_m,max,sat_	0.99863	0.00767 decade	750
*V*_th,sat_	0.99935	0.00937 V	750
Normalized drain-bias threshold shift	0.96954	0.17626 mV/V	750
SS, linear bias	0.87910	0.26228 mV/dec	695
SS, saturation bias	0.88473	0.24784 mV/dec	693

**Table 3:** Source-model claim outcomes after S2DS round-trip evaluation.

Target	Mean Absolute Error	Maximum Absolute Error	Source-Model Claim Pass
log_10_ *I*_on,sat_	0.00923 decade	0.03351 decade	8/9
log_10_ *g*_m,max,sat_	0.00957 decade	0.03123 decade	8/9
Δ*V*_th_/Δ*V*_DS_	0.11405 mV/V	0.26078 mV/V	9/9
*V*_th,sat_	0.01162 V	0.01628 V	7/9
All four targets	-	-	6/9

## Data Availability

The raw data and supporting code underlying the conclusions of this article are maintained in a version-controlled private GitHub repository and will be made available by the corresponding author upon reasonable request.

## Supplementary Materials

The following supporting information can be downloaded at: https://www.mdpi.com/article/10.3390/mi17091070/s1, Figure S1: Parameter-specific nearest-neighbor ambiguity analysis for the sealed 750-device independent test set.
