# Supplementary material for: Uncertainty-Aware Machine Learning for Compact-Model-Based Design and Identifiability Analysis of WSe2 p-Channel Transistors
Source: Micromachines (Basel). 2026 Sep 9;17(9):1070. doi: 10.3390/mi17091070 (PMC13609425; doi:10.3390/mi17091070)
Supplement: Supplementary file 1 [file micromachines-17-01070-s001.zip › micromachines-4516302-supplementary.pdf]

# Supplementary Materials

## Uncertainty-Aware Machine Learning for Compact-Model-Based Design and Identifiability Analysis of $WSe_2$ *p*-Channel Transistors

Zhengran He*, Kyeiwaa Asare-Yeboah, Meng Su, and Jie Zhao*

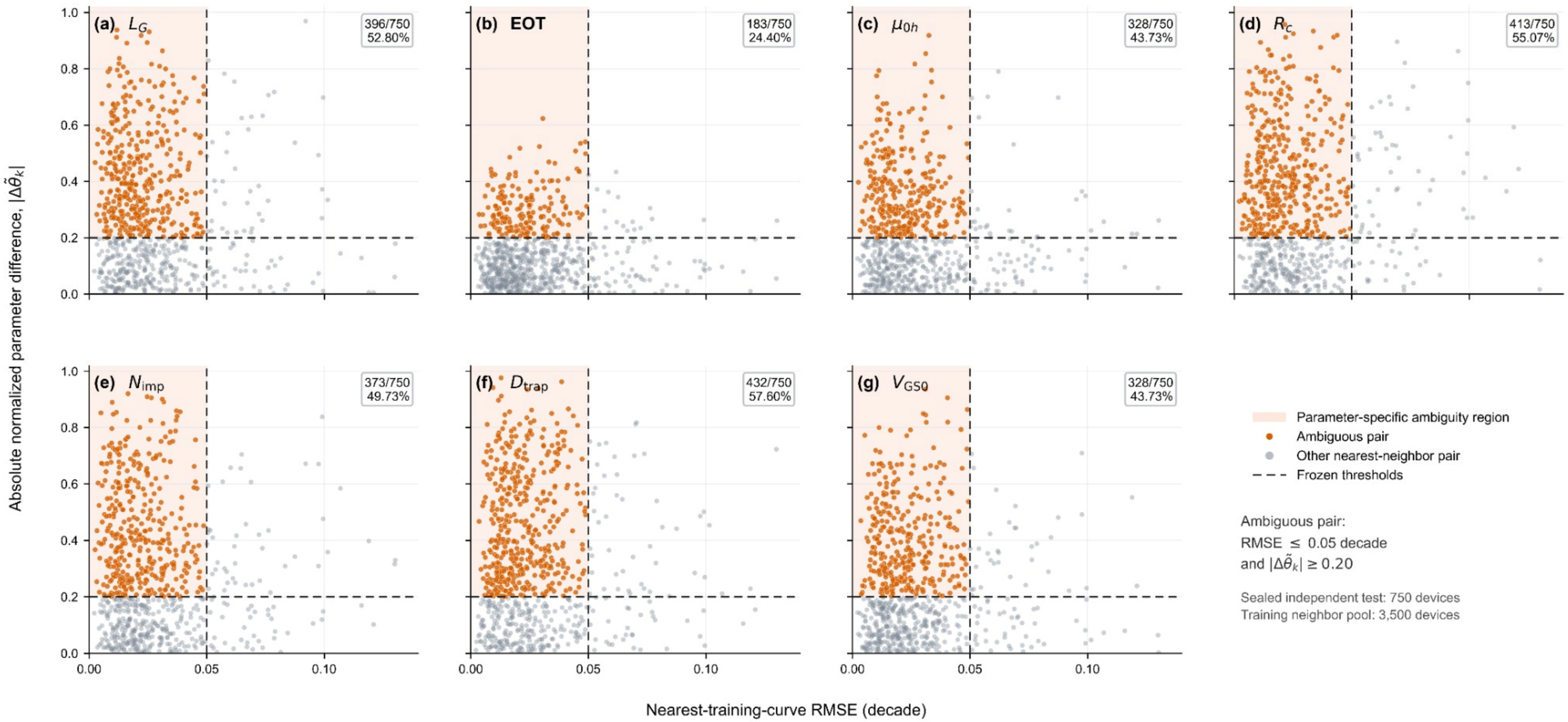


**Supplementary Figure S1.** Parameter-specific nearest-neighbor ambiguity analysis for the sealed 750-device independent test set. Panels (a–g) show the absolute normalized parameter difference between each test device and its nearest neighbor in the 3,500-device training ensemble as a function of nearest-training-curve RMSE for $L_G$, EOT, $\mu_{0h}$, $R_c$, $N_{imp}$, $D_{trap}$, and $V_{GS0}$, respectively. A pair is classified as ambiguous for parameter k when the curve RMSE is no greater than 0.05 decade and the absolute normalized parameter difference satisfies $|\Delta\tilde{\theta}\_k| \geq 0.20$. Orange points denote parameter-specific ambiguous pairs, and the shaded region indicates the corresponding ambiguity criterion. The resulting ambiguity rates are 52.80%, 24.40%, 43.73%, 55.07%, 49.73%, 57.60%, and 43.73%, respectively.
